# Numerical simulation of the effect of internal hole defect size on the mechanical properties of limestone

**DOI:** 10.1371/journal.pone.0275626

**Published:** 2022-10-07

**Authors:** Handong Liu, Shuai Liu, Yawen Zhao, Jialiang Wang, Chao Zheng, Zhiguo Xia, Guang Zheng

**Affiliations:** 1 Henan Key Laboratory of Geomechanics and Structural Engineering, North China University of Water Resources and Electric Power, Zhengzhou, China; 2 College of Geosciences and Engineering, North China University of Water Resources and Electric Power, Zhengzhou, China; 3 School of Civil and Transportation Engineering, Henan University of Urban Construction, Pingdingshan, China; 4 School of Mining Engineering, University of Science and Technology Liaoning, Anshan, China; 5 MOE Key Laboratory of Impact and Safety Engineering, Ningbo University, Ningbo, China; Sapienza University of Rome: Universita degli Studi di Roma La Sapienza, ITALY

## Abstract

To better understand the effect of the size of hole defects on the mechanical properties of a rock mass, the two-dimensional particle flow discrete element code (PFC2D) is applied to establish rock mass models with single circular hole defects of different diameters. Uniaxial compressive strength (UCS) tests are conducted on each model by only taking the defect size (area) as a variable. This study analyzes each model’s stress-strain, contact force chain, crack evolution, meso-damage and failure, and mechanical properties. The results showed that with the size enlargement of the circular hole defects, each model’s UCS and elastic modulus gradually decrease, and the defect size is negatively correlated with the mechanical strength of the rock samples. The size of the hole defects affects the entire process of contact force chain and crack evolution. The larger the aperture dimension of the circular hole defects in each model, the greater the concentration degree of the contact force chain, the earlier the crack initiation, and the higher the degree of crack coalescence in the post-peak stage. The number of cracks decreases as the hole size increases, and the model is more prone to failure. Rock models’ strength and failure characteristics with different numbers and arrangements of hole defects are discussed under the same defect area condition.

## Introduction

Rock masses under natural conditions have many defects, such as holes and microcracks. The size and geometric distribution of these defects directly affect the intrinsic properties and engineering stability of the rock masses containing them [1–5]. Circular holes are a common and typical rock defect, and it is valuable to study the impact of circular hole defects of different sizes on rock damage and mesomechanics. It is essential to investigate the effects of hole defects on the macroscopic deformation and failure properties of rock masses and control the potential harm of the hole defect rock masses.

The study of defective rock masses is one of the ongoing focuses of international rock mechanics. Many studies have been conducted on the stress calculation, failure and strength variation characteristics, meso damage, and crack evolution of defective rock masses [6, 7]. Most of the defect types in these investigations are regular holes (rectangular, oval, and others) and joints [8–10], with a logical progression to combinations of defects, such as holes and fissures [11, 12]. He et al. [13] analyzed the mechanical behavior and energy variation of red sandstones containing one, two, and three circular holes. Sammis and Ashby [14] utilized theoretical calculations and laboratory tests to examine the interaction between new cracks and the surface of porous samples, and established a theoretical damage mechanics model for brittle porous solids under multiaxial compression. Lin et al. [15] investigated the crack evolution and damage morphology of granitic material with multiple circular holes through uniaxial compression and modified the Sammis and Ashby cracking model to predict the peak stress in rock samples. They concluded that the number of holes has the greatest influence on the peak stress. Zhou et al. [16] and Du et al. [17] conducted uniaxial compressive strength (UCS) tests on samples with double circular holes, and investigated the strength, fracture mode, and crack evolution characteristics of the samples with different circular hole spacings, inclinations, and filling conditions. Tang et al. [18] carried out numerical experiments on rock samples with single-hole, three-hole, and pore-like defects and simulated the observed splitting failure phenomenon in brittle solids. Huang et al. [19] performed numerical simulations and laboratory tests on granite samples containing three non-coplanar holes to analyze the crack development and damage mechanism of the samples. Ma et al. [20] compared the influence of the sequence and arrangement of circular hole defects on the mechanical properties of concrete specimens, with emphasis on the crack evolution mechanism. Xia et al. [21] and Gui et al. [22] addressed the impact of circular hole defects and other shape defects on the mechanical features and damage modes of rock masses using PFC2D and mixed continuous discrete element methods, respectively.

In studies of the effect of the size of defects, the focus has been on the influence of rock mass size on the mechanical properties of the defective rock mass. Bahrani and Kaiser [23] applied a grain-based distinct element model (GBM) to study the effect of rock sample size on the strength of a rock mass with defects and perform a size effect analysis. Tang et al. [24] and Lyu et al. [25] analyzed the relationship between rock heterogeneity (initial defects) and the strength size effect of rock, and proposed that the heterogeneous characteristics of rock samples are significantly affected by the size of defects. Wong et al. [26] conducted a series of physical and numerical uniaxial compression tests on specimens with a single circular hole defect and various height-to-diameter ratios. Similar to Wong et al. [26], Gui et al. [27] comprehensively studied the influence of circular hole defects on rock mechanical behavior. Their study included changing the diameter of the single circular holes and the size of the test pieces, and they also analyzed samples with a mixed distribution and arrangement of various multiple circular holes of different sizes. Jing et al. [28] used a new method for testing damaged rock samples and explored the effect of defect size for rock samples on uniaxial strength attenuation using PFC2D.

The above studies have provided rich results on the size effect of defect on the mechanical features of defective rock masses. They also resulted in identifying and formulating scientifically reliable research methods, such as numerical simulation methods like PFC2D. However, previous investigations of circular hole defects focused on the number of circular holes and their arrangement or a comparison with other defects. However, studies that vary the size of individual circular hole defects are rare. Although varying the number of hole defects involves changing the size of the defect area, the location and distribution of the holes also change, and the influencing factors are not unique. Where size effects are concerned, previous studies tended to change the overall size or height-to-diameter ratio of the samples or the concentrated area of the defects in the rock mass; they have paid less attention to the impact of the size of the same defect on the damage and failure of rock masses and their meso mechanical properties. Accordingly, a study that considers only the defect size (area) as a variable can better illustrate the variation in the mechanical properties of a rock mass with different hole defect sizes. This research focuses on the damage, failure, and meso-mechanical properties of limestone containing a single circular hole defect with various diameters using the PFC2D code.

## Numerical simulation parameter determination and model establishment

### Determination of simulation parameters

**Fig 1:**
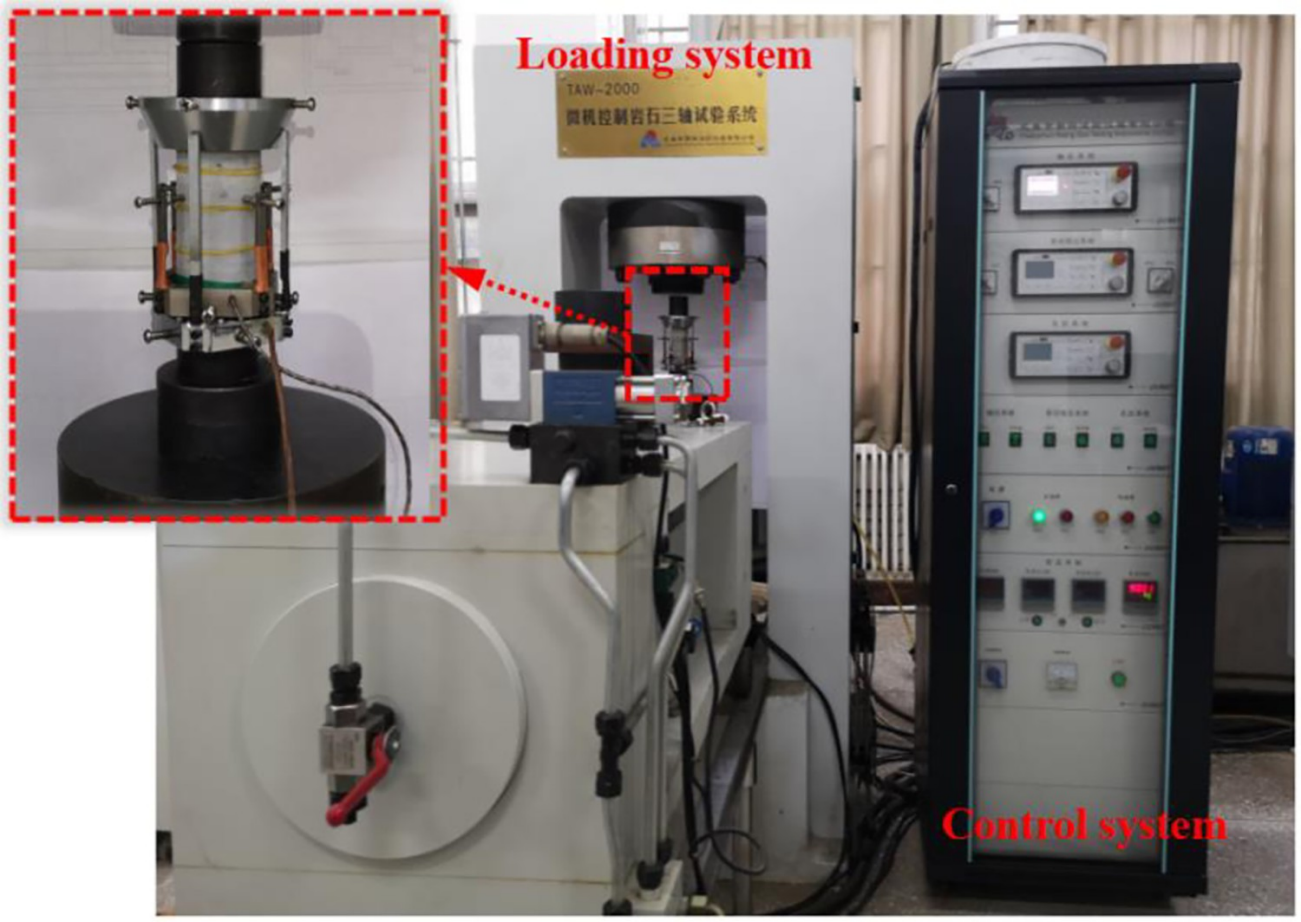
Test equipment.

The tight and fresh limestone samples were first subjected to laboratory tests to determine their physical and mechanical parameters. The UCS tests were carried out using a TAW-2000 rock triaxial test system, and the test instrument is shown in Fig 1. According to the ISRM test specification [29], standard cylindrical rock samples with a height to diameter ratio of 2:1 (100 mm×φ50 mm) were used for the uniaxial compression tests. The loading was controlled with a deformation of 0.001 mm/s.

**Fig 2:**
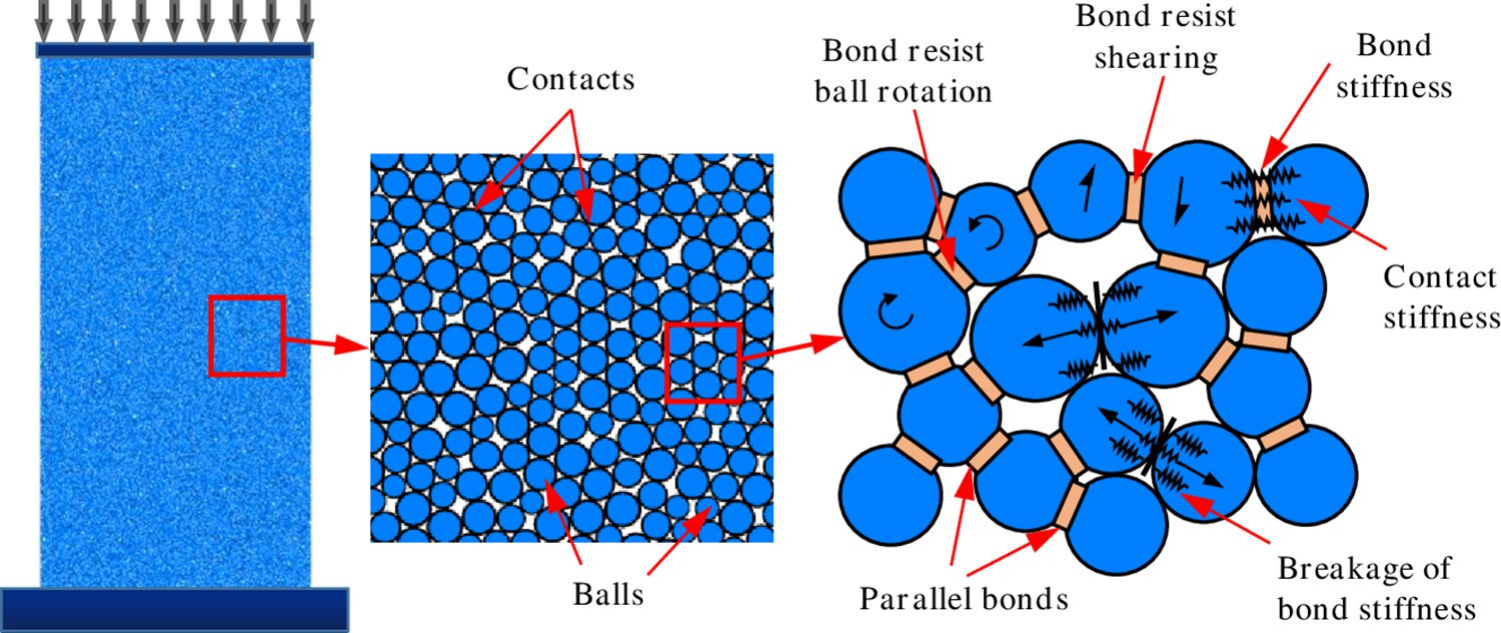
Typical numerical model and the parallel bond contact in PFC2D.

Compared to macroscopic laboratory tests, the PFC2D code is suitable for analyzing the meso characteristics of rocks, such as crack evolution, stress concentration area, and others, during its progressive failure. It is a very reliable numerical simulation method in the field of rock mechanics [30–32]. Several researchers also studied the meso-mechanical properties of solid media based on continuum mechanics [33–35]. The cracking-particle method, which can deal with complex fracture problems and is easily calibrated, is advanced and reliable [36, 37]. However, the basic unit particles used in PFC2D are circular (spherical), and a contact model connects the interaction between particles. The parallel bonding model can transmit force and moment simultaneously, and the contact range is large, which is suitable for simulating rock materials [38, 39]. A typical numerical model of uniaxial compression in the PFC2D code and a schematic representation of the parallel bond contact are shown in Fig 2.

**Fig 3:**
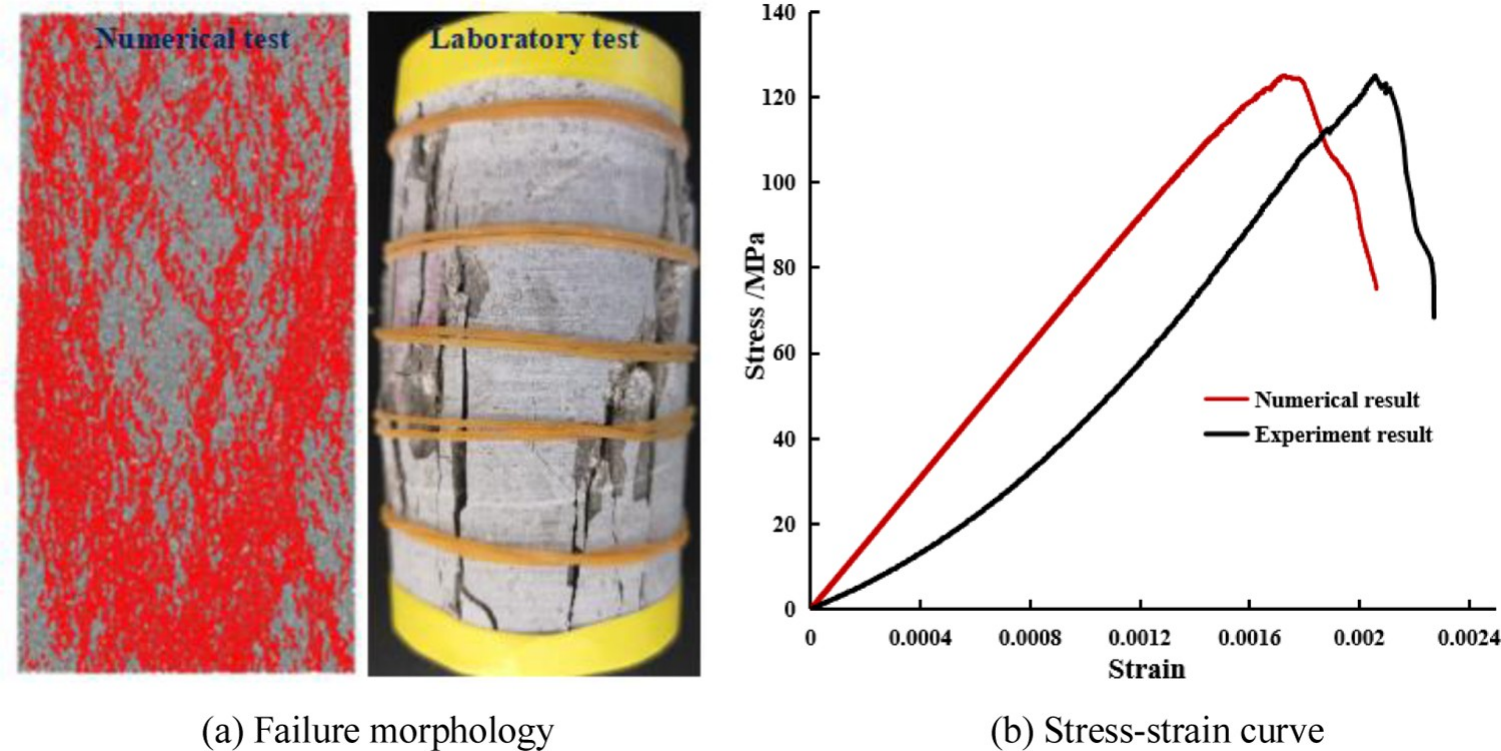
Verification of the experimental and numerical results. (a) Failure morphology, (b) Stress-strain curve.

Before establishing the numerical model for simulation, it is necessary to build the same model used in the laboratory test. The numerical simulation and laboratory test results are compared to ensure that the two test results are consistent [40, 41]. The stress-strain curves derived from the PFC2D program were calibrated using the same test methods and loading conditions as in the laboratory tests. Then, the microscopic parameters suitable for PFC2D simulation were obtained, as shown in Table 1. The failure morphology and uniaxial stress-strain curve of the rock sample’s model obtained from the experiment and the numerical comparison are shown in Fig 3. The failure patterns of the experimental samples and the numerical samples are similar, and their peak strength and deformation modulus are the same, indicating that the microscopic parameters of the PFC2D model meet the reliability requirements of the simulation test.

**Table 1:** Microscopic parameters of rock.

Parameters	Rock (value)
Minimum particle diameter (mm)	0.2
Particle diameter ratio	1.5
Grain density (g/cm^3^)	3.05
Contact modulus of the particle (GPa)	36.21
Contact bond gap (mm)	0.05
Porosity	0.1
Parallel bond friction angle (°)	42.5
Parallel bond tensile strength (MPa)	10.95
Normal critical damping ratio	0.5
Parallel bond cohesive force (MPa)	52.23

**Fig 4:**
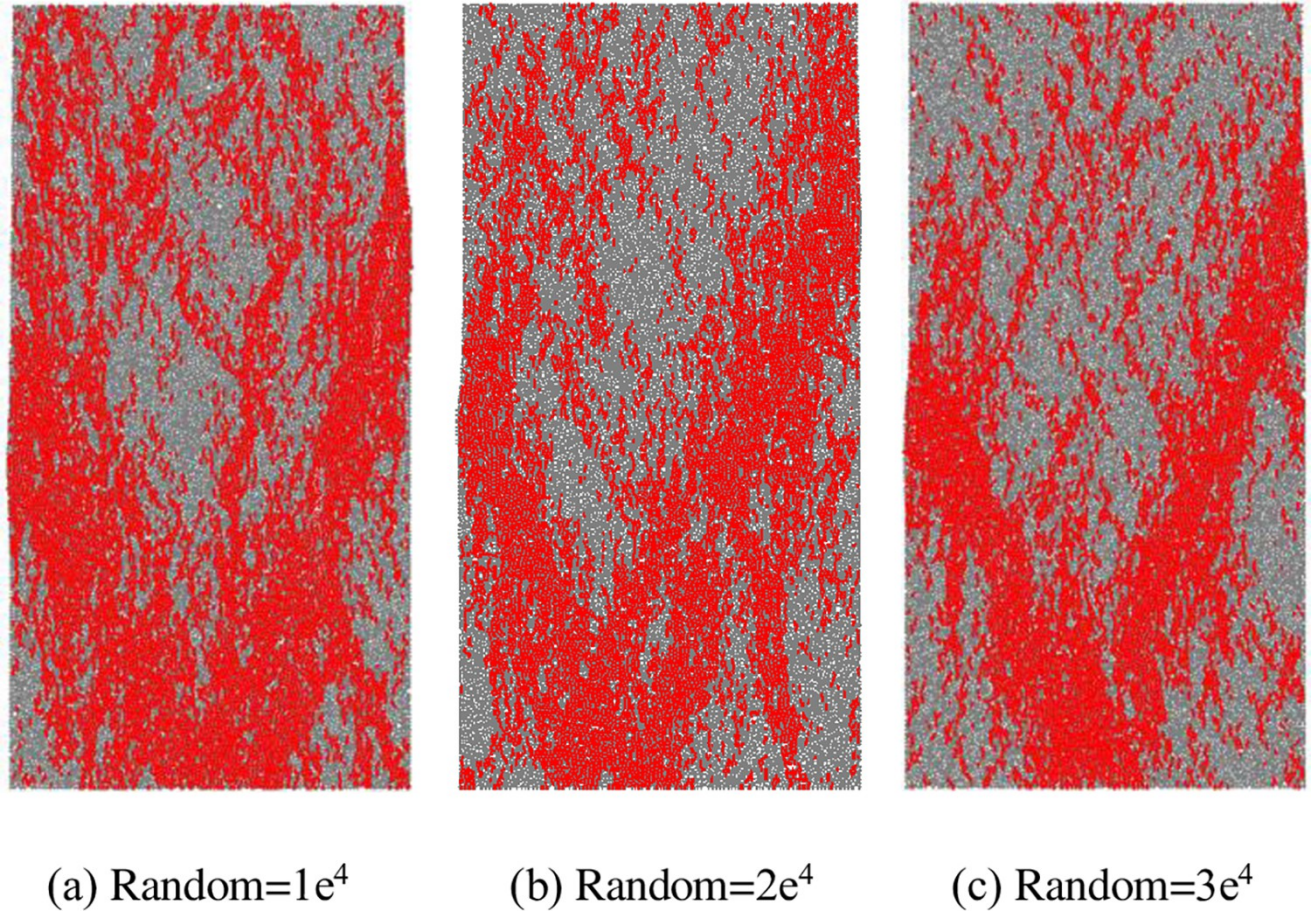
Failure modes of the intact model under different random seeds. (a) Random = 1e4, (b) Random = 2e4, (c) Random = 3e4.

In order to verify the applicability and scientificity of meso-parameters, it is necessary to analyze the uncertainty of particle distribution in the model [42]. In PFC numerical simulation, random seeds are usually used for verification [31, 43]. Therefore, before the numerical simulation test, the failure mode and mechanical properties are analyzed by setting different random seeds of the complete model. In Fig 3, the random seed of the numerical model is 1e^4^, and then the random seed is changed to 2e^4^ and 3e^4^, respectively. The mechanical parameters and failure modes of the complete model are shown in Table 2 and Fig 4 under different random seed conditions. The elastic modulus is the same in the models with different random seeds, and the peak stresses are also close. The meso-parameters in the PFC2D model have high reliability and applicability.

**Table 2:** Peak stress and elastic modulus under different random seeds.

Parameters	Rock sample	PFC2D numerical models		
		Random = 1e^4^	Random = 2e^4^	Random = 3e^4^
Peak stress (MPa)	125.04	125.05	122.98	123.71
Elastic modulus (GPa)	77.10	77.11	77.11	77.11

### Establishment of the numerical models

**Fig 5:**
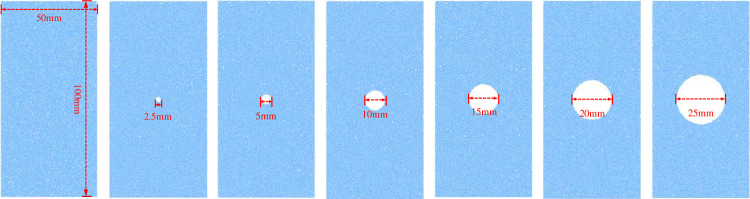
Numerical models: The diameters of the circular hole defects from left to right are 0, 2.5, 5, 10, 15, 20 and 25 mm.

The numerical models containing circular hole defects of different sizes were established using the parallel bond model. The height and diameter of each numerical model were set to 100 mm and 50 mm respectively, and each model contained only a single circular hole defect. The diameters of the circular holes in each model were 0 mm (intact model), 2.5 mm, 5 mm, 10 mm, 15 mm, 20 mm, and 25 mm, and the circular hole was located in the model’s center. An illustration of each of the numerical models is shown in Fig 5.

## Analysis of simulation results

### Stress-strain characteristics of rock samples with different defect sizes

**Fig 6:**
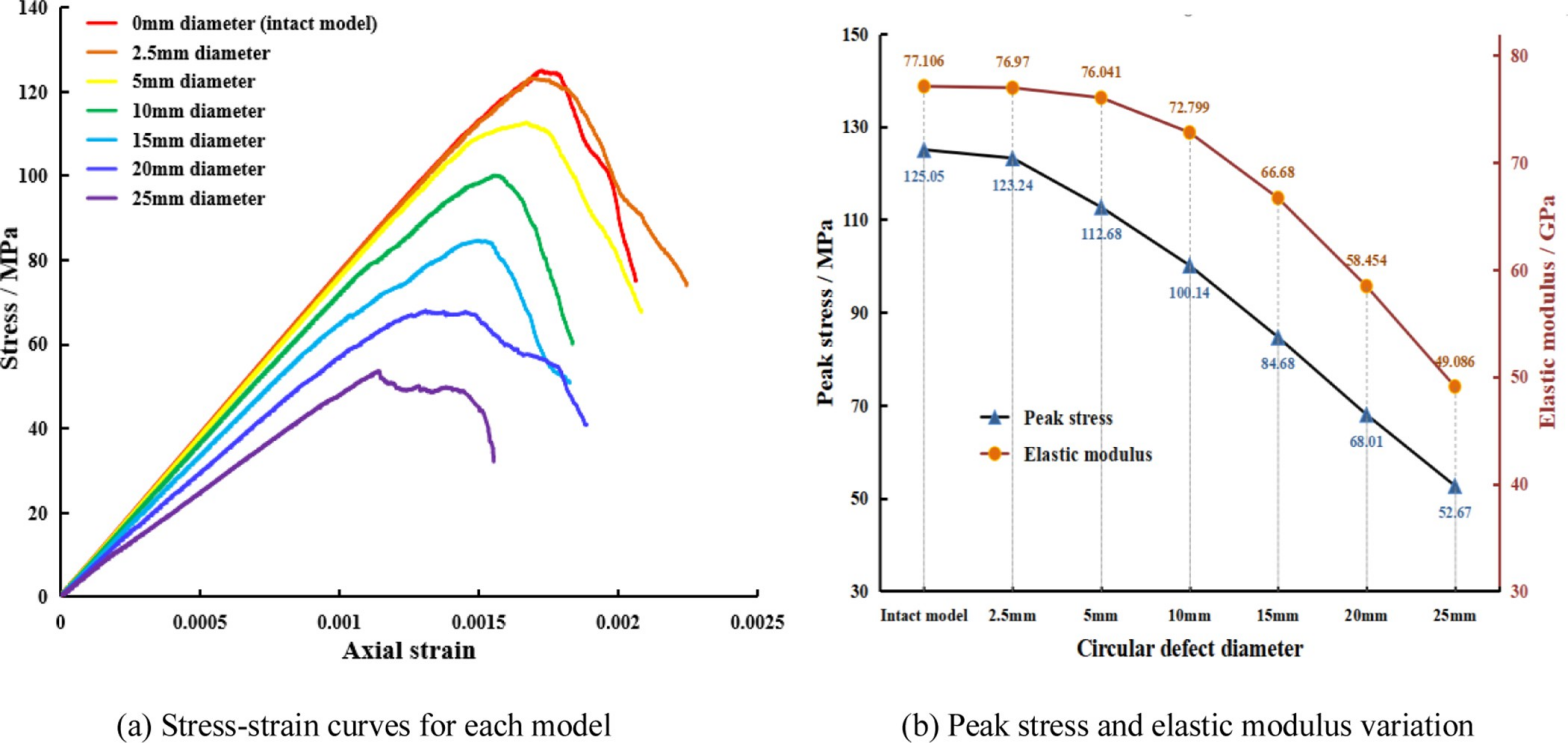
Stress-strain curves, elastic modulus, and peak stress curves of each model. (a) Stress-strain curves for each model, (b) Peak stress and elastic modulus variation.

The stress-strain curves of each model under uniaxial compression are shown in Fig 6(A), and the peak stress and elastic modulus changes are indicated in Fig 6(B). Fig 6 revealed that the peak stress and elastic modulus of the intact rock sample’s model are the largest, at 125.05 MPa and 77.11 GPa, respectively. When the defect diameter is 2.5 mm, the effect on the stress-strain curve of the model is not evident because the defect size is 1/20 of the bottom diameter of the rock sample. However, this is visible as the peak stress and elastic modulus are slightly reduced (by 1.81 MPa and 0.14 GPa, respectively). This indicates that even with relatively small defect sizes, there is still a degrading effect on the mechanical properties of the rock sample. With the increase in the circular defect’s diameter, the peak stress and elastic modulus of the rock samples decrease significantly. The peak stresses at the defect sizes of 1/10, 1/5, 3/10, 2/5, and 1/2 of the bottom diameter of the rock sample are 112.68 MPa, 100.14 MPa, 84.68 MPa, 68.01 MPa, and 52.67 MPa, respectively, and the elastic moduli are 76.04 GPa, 72.80 GPa, 66.68 GPa, 58.45 GPa, and 49.09 GPa, respectively. The UCS and elastic modulus show a gradual downward trend. Compared with the intact model, when the defect size reaches half of the rock sample’s bottom diameter, the UCS and elastic modulus decrease by 57.88% and 36.34%, respectively. Fig 6(A) shows that the peak strain of each model is gradually reduced as the defect size gradually increases. This indicates that the larger the defect size, the progressively lower the amount of deformation the rock samples can withstand, so the rock sample is damaged earlier.

The above analysis shows that circular hole defects have a direct effect on the mechanical properties of rock samples. If there are circular hole defects, regardless of the size of the defect, the mechanical properties of the rock sample will deteriorate. As the size of the circular hole increases, the UCS and elastic modulus gradually decrease, and the size of the defect is negatively correlated with the mechanical strength of the rock sample.

### Contact force chain and stress field analysis

**Fig 7:**
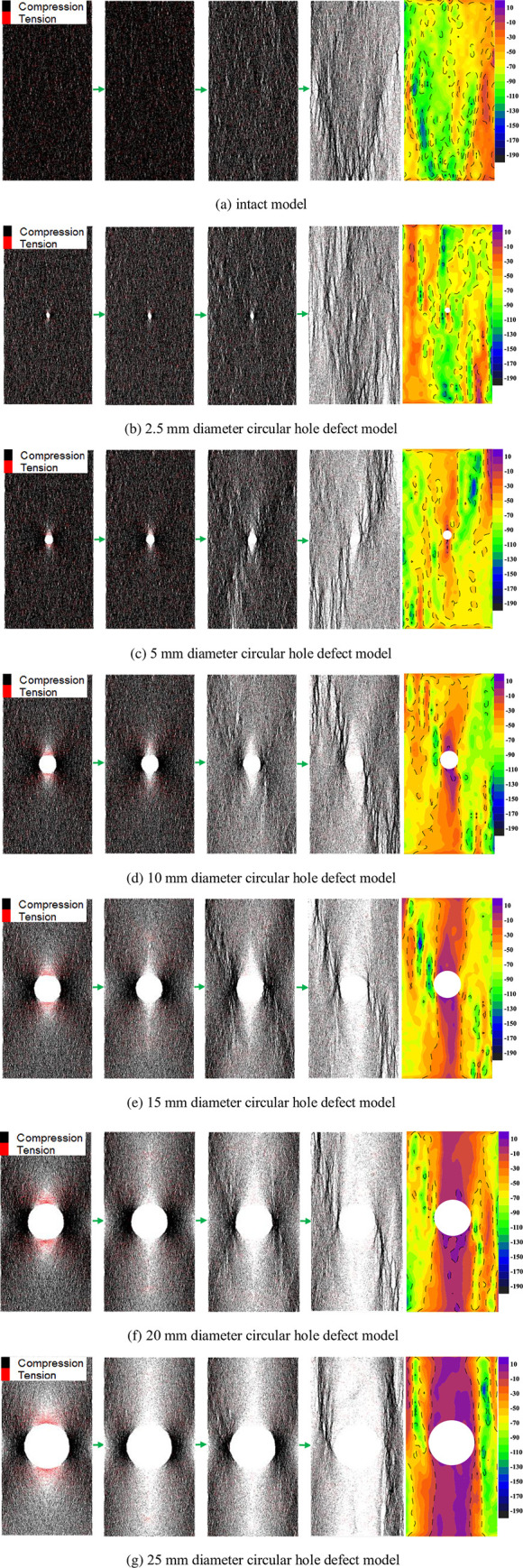
Evolution of the contact force chain and distribution of the post-peak stress field in each model: The corresponding stress levels from left to right are 10% of the UCS, 50% of the UCS, 100% of the UCS, and 60% of the UCS in the post-peak failure stage. (a) intact model, (b) 2.5 mm diameter circular hole defect model, (c) 5 mm diameter circular hole defect model, (d) 10 mm diameter circular hole defect model, (e) 15 mm diameter circular hole defect model, (f) 20 mm diameter circular hole defect model, (g) 25 mm diameter circular hole defect model.

Based on the UCS of each model, the evolution characteristics of the contact force chain of each model were analyzed at the stress levels of 10%, 50%, and 100% of the peak stress and 60% of the peak stress in the post-peak stage, respectively. The corresponding vertical stress field distribution characteristics in the post-peak stage are also displayed correspondingly (rightmost pictures). The red force chains represent tensile stresses, and the black force chains represent compressive stresses. The more dense and darker color of the force chains in the parallel contact force distribution diagram, the greater the stress concentration. The evolution of the contact force chains and the distribution of the vertical stress field in the post-peak stage of each model are shown in Fig 7.

In Fig 7, the contact force chains of the complete model are relatively evenly distributed before the peak strength, with the tensile and compressive force chains interacting. When the peak stress is reached, the contact force chain appears to be in a relatively dispersed state. In the post-peak stage, the contact force chain is relatively loose on the whole, and the main concentrated area of the compressive force chain is formed near the failure position of the rock sample. Also, a V-shaped distribution is formed roughly at the center of the bottom surface of the model. The distribution characteristics of the vertical stress field after the peak are consistent with the distribution of the force chain. In the model with a circular hole defect size of 2.5 mm in diameter, a little concentration of compressive force chains can be seen on both sides of the circular hole before the peak stress, with a more even and dense distribution of tensile force chains in other areas of the model. In the post-peak stage, the concentrated area of the compressive force chain and the vertical stress is mainly in the vertical center of the model. In the defective rock mass models with hole diameters of 5 mm, 10 mm, 15 mm, 20 mm, and 25 mm, before the peak stress, the contact pressure chain concentration areas of each model are similar, mainly concentrated near the circular hole defect. The compressive force chain is mainly concentrated on the left and right sides of the circular hole, and the tensile force chain is mainly concentrated near the upper and lower sides of the hole. When the peak stress is reached, there is still an obvious compressive force chain concentration phenomenon on the left and right sides of the circular hole in each model, and a compressive force chain distribution trend similar to an "X" shape is formed in the model with the circular hole as the center. The tensile force chain near the side disappears, almost forming a blank area of the force chain. In the post-peak stage, in each model, a diagonal compressive force chain concentration area mainly forms along the circular hole defect, and a sparse force chain area forms in the vertical middle area of the model, which also indicates the main failure mode of the model. The vertical stress field of each model with a circular hole defect diameter of 5–25 mm in the post-peak stage is mainly manifested in the form of a low-stress area or a low tensile stress area in the vertical middle of the model. The large and vertical stress concentration areas are primarily on both sides of the model, and the range of the concentration areas is consistent with the range of the stress chain concentration area.

Comparing the evolution of the force chain of each model shows that the circular hole defect significantly influences the evolution of the force chain inside the rock sample. At the 10% peak stress level, with the increase in the defect size of the circular hole, the larger the defect size, the greater the concentration of the force chain near the circular hole, and the larger the range. For example, in the 5 mm diameter circular hole model, the concentration of force chains near the hole is only slightly increased, and the overall force chains in the model are more evenly distributed. However, in the 25 mm diameter circular hole model, the force chains are mainly concentrated near the hole, and the force chains in the other areas of the model are relatively sparse. This phenomenon affects the concentration degree of the force chain in the vicinity of the circular hole and persists up to the peak stress. The degree of concentration and distribution of force chains near the round hole is positively correlated with the size of the circular hole defect. The larger the circular hole defect, the greater the relative stress concentration and the more likely the rock sample will break, which is consistent with the negative correlation between the compressive strength and the defect size in each model. Up to the post-peak stage, the larger the defect size in the middle of the model, the sparser the distribution of force chains above and below the circular hole, and the greater the extent of the low-stress region above and below the circular hole in the middle of the vertical stress field. In general, although the force chain distribution patterns are similar in the models with differently-sized circular hole defects, the defect size controls the concentration and distribution range of the force chain. The concentration and distribution range of the contact force chain is positively correlated with the size of the circular hole defect.

### Crack initiation stresses and strains of each model

**Fig 8:**
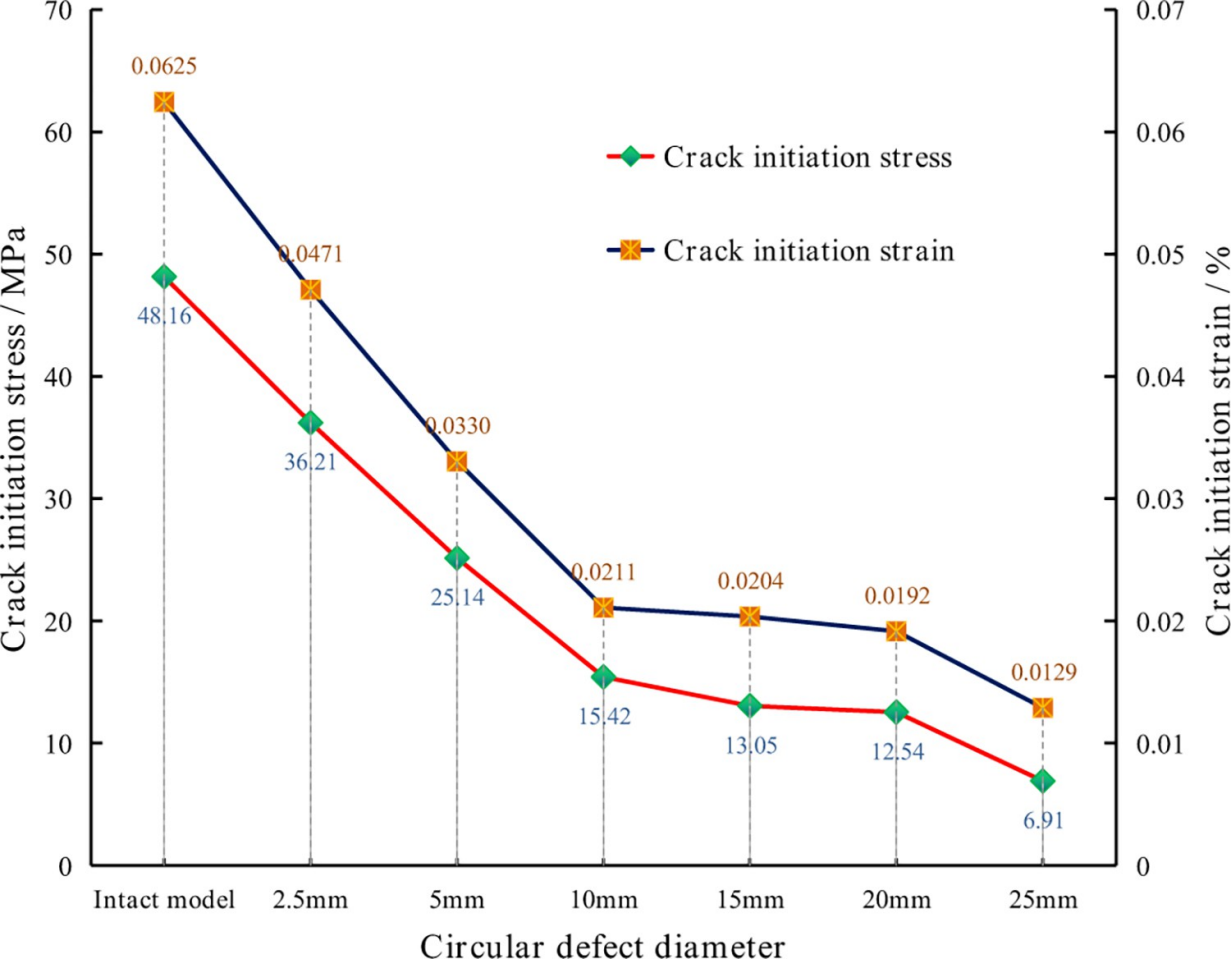
Crack initiation stress and crack initiation strain in each model.

Tight rock samples will not generate new cracks until they are uniaxially loaded to a certain extent. At this time, the corresponding stress state is called the crack initiation stress, which is also an essential characteristic of rock strength [44–46]. In the PFC2D code, the crack initiation stress can be determined by monitoring through the microcrack method [47, 48]. Then, the influence of the size of the circular defect on the crack initiation stress of the rock sample is determined. The crack initiation stress and strain in each model are shown in Fig 8.

Fig 8 indicates that the crack initiation stress and strain of the intact rock sample are about 48.16 MPa and 0.0625%, respectively, and the stress level at the crack initiation point is about 38.52% of the peak stress. The crack initiation stress and strain of the models with the 2.5 mm, 5 mm, and 10 mm diameter circular defects all decrease rapidly, and the decreasing trend is almost linear with the increase in the size of the circular hole defects. This indicates that presence of the circular hole defects significantly reduces the crack initiation stress in the rock. In the models with the 15 mm and 20 mm diameter circular hole defects, the downward trend of the crack initiation stress and strain becomes slow, and in the model with the 25 mm diameter circular hole defect, there is an accelerated downward trend again. The crack initiation stress value of each model, the percentage of the UCS, and the relative decline rate are shown in Table 3. In general, with the increase in the circular hole defect size, the model’s crack initiation stress gradually decreases, and the rock sample is more prone to damage and deformation.

**Table 3:** Crack initiation stress value, percentage of the UCS, and the relative reduction rate.

Circular defect models	Crack initiation stress (MPa)	Percentage of UCS	Relative reduction rate
Intact model	48.16	38.52%	0%
2.5 mm diameter model	36.21	29.38%	24.81%
5 mm diameter model	25.14	22.31%	47.80%
10 mm diameter model	15.42	15.40%	67.99%
15 mm diameter model	13.05	15.41%	72.90%
20 mm diameter model	12.54	18.43%	73.97%
25 mm diameter model	6.91	13.12%	85.65%

### Analysis of crack evolution and failure mode

**Fig 9:**
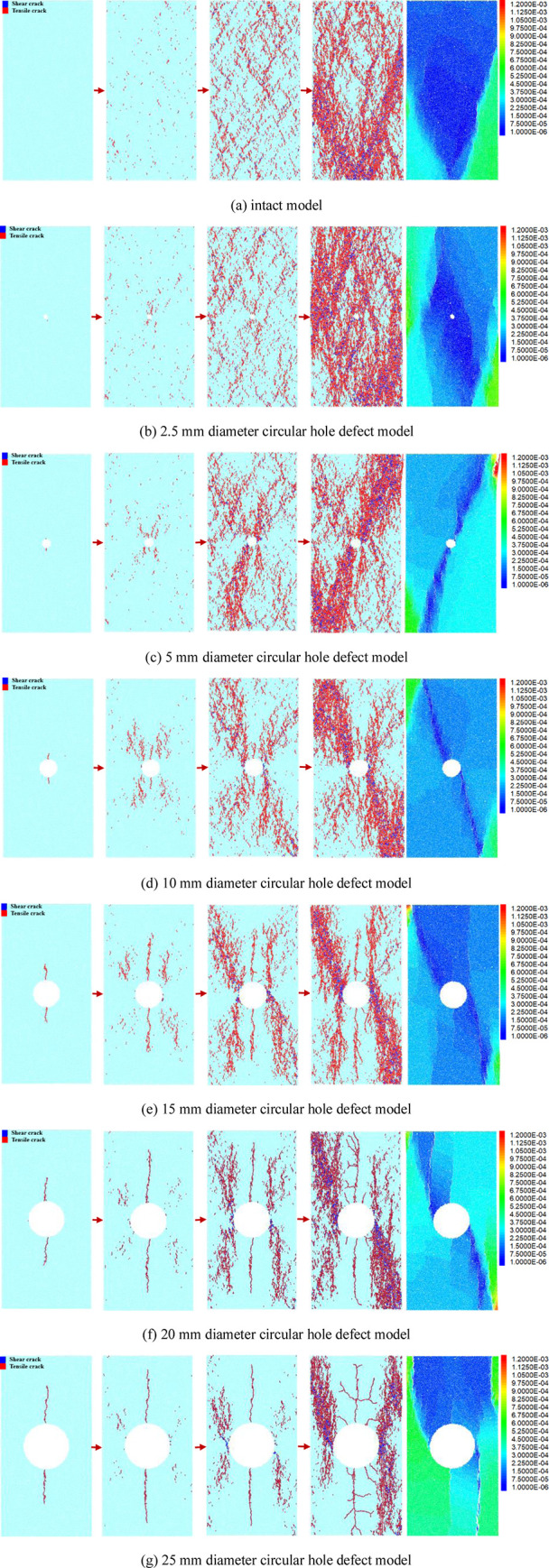
Crack evolution and post-peak displacement field distribution characteristics of each model: The corresponding stress levels from left to right are 30% of the UCS, 70% of the UCS, 100% of the UCS, and 60% of the UCS in the post-peak failure stage. (a) intact model, (b) 2.5 mm diameter circular hole defect model, (c) 5 mm diameter circular hole defect model, (d) 10 mm diameter circular hole defect model, (e) 15 mm diameter circular hole defect model, (f) 20 mm diameter circular hole defect model, (g) 25 mm diameter circular hole defect model.

Based on the UCS of each model, at the stress levels of 30%, 70%, 100% of the peak stress and 60% of the peak stress in the post-peak stage, to analyze the crack evolution characteristics of each model and the corresponding post-peak stage displacement field distribution characteristics. The crack evolution and post-peak displacement fields within each model are shown in Fig 9, where the blue cracks are shear cracks and the red ones are tensile cracks.

In Fig 9, at the 30% peak stress level, the intact model shows no crack generation. A crack has just been initiated in the model with the 2.5 mm diameter circular hole, and the crack initiation position is near the circular hole. In each model containing the 5 mm-25 mm diameter circular holes, cracks are generated at the upper and lower vertices of the circular holes, and as the size of the hole increases, the cracks expand outward along the upper and lower vertices of the hole, and the degree of crack coalescence is great. This shows that the larger the defect size under lower loading stress, the earlier the crack initiation in each model. At the 70% peak stress level, relatively scattered compression cracks are developed in the intact model. In each model with circular hole defects, the crack at the upper and lower vertices of the circular hole continues to expand. Except for the model with the 2.5 mm diameter circular hole where new cracks are scattered within the model, there is only a slight concentration of cracks at the circular hole. All the other models with hole defects have cracks mainly concentrated near the hole, except for the vertical cracks developed at the top and bottom vertices of the circular hole which continue to expand by some amount. The primary new cracks are distributed on the left and right sides of the circular hole and appear in a roughly X-shaped distribution with the circular hole at the center. At the peak stress, many scattered cracks are developed in the intact model and the model with the 2.5 mm diameter circular hole. In the other models with circular hole defects, the concentrated cracks with the original X-shaped distribution continue to expand and develop, and more shear cracks also appear. In contrast, the tensile cracks at the top and bottom vertices of the circular hole develop more slowly, with only a small amount of extension. At post 60% of the peak stress, each model becomes damaged, the number of cracks in each model reaches the maximum, and the number of shear cracks develops rapidly in the post-peak stage.

**Fig 10:**
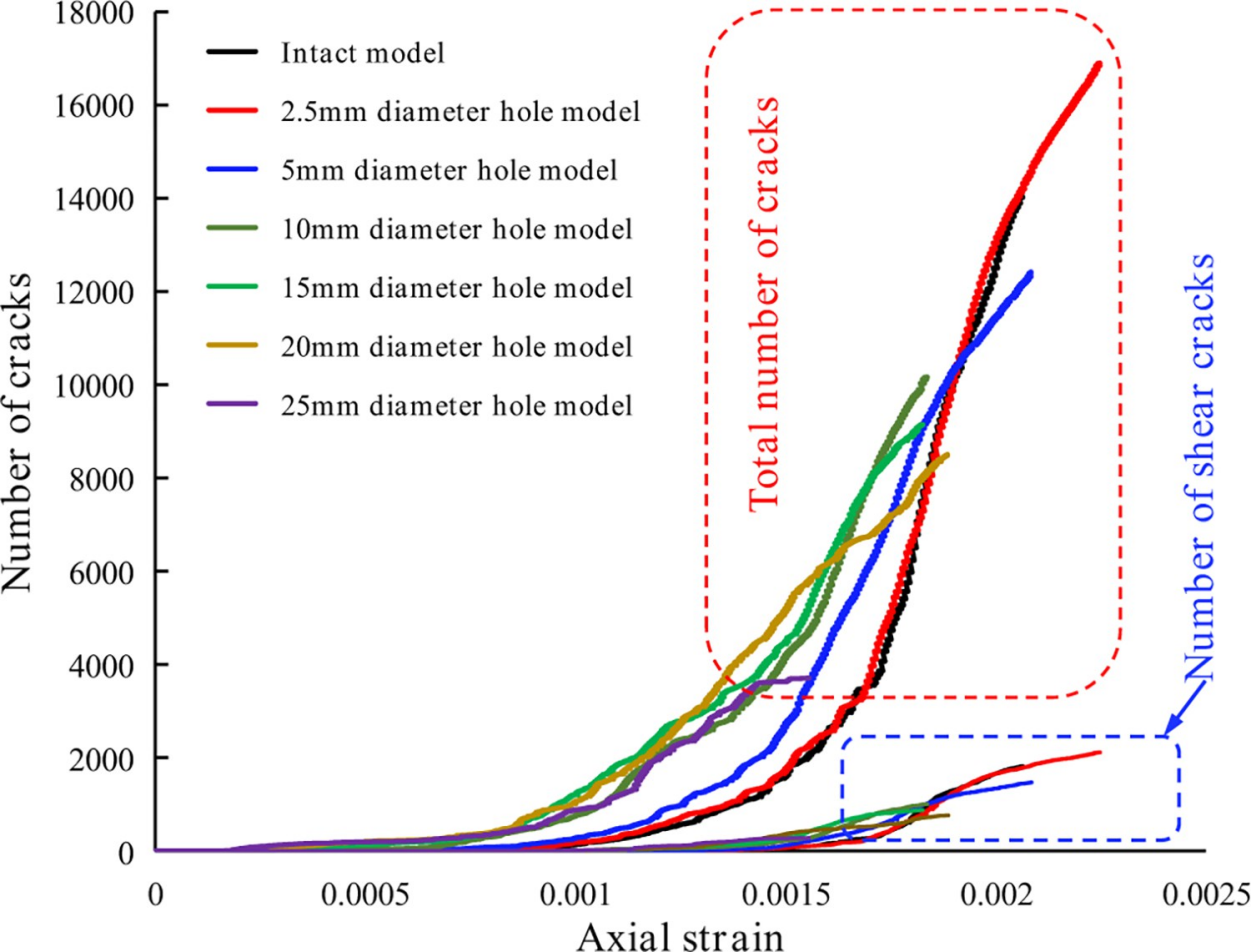
Variation curves of the total number of cracks and the number of shear cracks for each model.

The area through cracks is the central area of model failure, which are consistent with the distribution characteristics of the displacement field of each model. The intact model mainly undergoes conical splitting failure, while the model with the 2.5 mm diameter round hole mainly undergoes oblique splitting failure on the upper left and lower right side of the rock sample. In other circular hole diameter models, the area along the diagonal line of the model with the circular hole as the center is the main failure range. With the increase in the defect size of the circular hole, the degree of crack coalescence at the diagonal area of the rock sample is higher, and the vertical cracks at the upper and lower vertices of the circular hole gradually penetrate the rock sample. To a certain extent, the number of cracks can represent the degree of fragmentation and the difficulty of failure of the rock sample. Fig 10 shows the variation curves of the total number of cracks and the number of shear cracks for each model, and Table 4 shows the number of cracks in each model at post 60% of the peak stress. The crack counts show that the model with the 2.5 mm diameter round hole has more cracks than the intact model. However, all the other models have fewer cracks than the intact model, and the total number of cracks and the tensile and shear cracks decrease gradually as the size of the hole defect increases.

**Table 4:** Number of cracks developed in each model.

Circular defect models	Total number of cracks	Tensile crack	Shear crack
Intact model (0 mm diameter)	14145	12325	1820
2.5 mm diameter model	16902	14789	2113
5 mm diameter model	12420	10950	1470
10 mm diameter model	10166	9163	1003
15 mm diameter model	9144	8270	874
20 mm diameter model	8507	7745	762
25 mm diameter model	6881	6306	575

The above analysis shows that the size of the circular hole defect influences the entire process of crack evolution in terms of crack initiation, extension, and coalescence. This results in significant differences in the development, number, distribution range, and failure path of the cracks in each model. As the size of the circular hole defect increases, cracks at the top and bottom vertices of the circular hole start to develop earlier. In addition, they develop in greater numbers and have a higher degree of coalescence after model failure. The total number of cracks developed and the number of tensile and shear cracks in each model in the post-peak stage tend to decrease gradually, indicating that the larger the size of the circular hole defect, the more likely the model is to fail.

## Discussion

This study establishes numerical models of limestone with single circular hole defects of different sizes using the PFC2D code. Under the guarantee of the uniqueness of the defect size variable, the changes in the stress and strain of each model with different defect sizes, as well as their influence on the meso-mechanical characteristics, such as contact force chain and crack evolution, were comprehensively analyzed. However, defects in rock masses are often not single, and the same kinds of defects are often scattered in a rock mass. Therefore, it is also necessary to consider the number and arrangement of circular hole defects to analyze the effect of defect size on the mechanical features of a rock mass.

**Fig 11:**
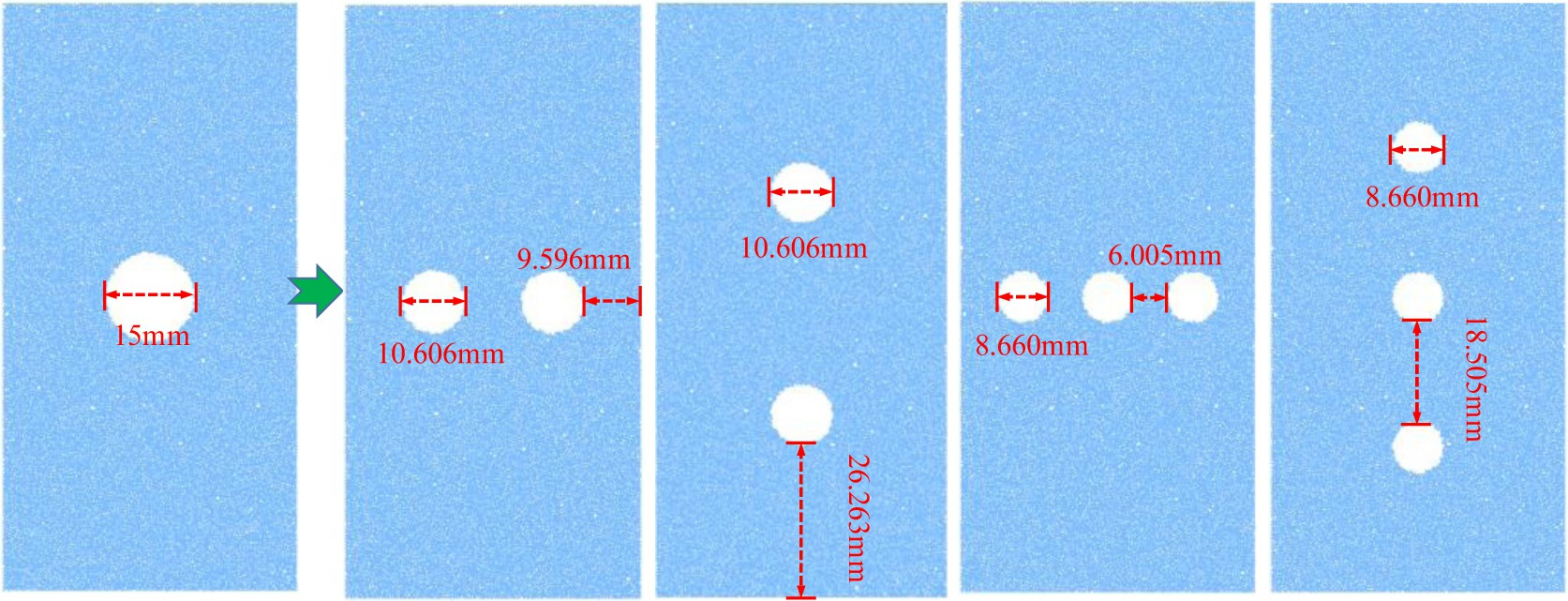
Comparison model of the number and arrangement of circular hole defects.

In previous similar studies, Wong et al. [26] and Gui et al. [27] considered the case of varying the circular hole diameter and the specimen size in specimens with single circular hole defects; they also considered the case of a mixed distribution of different, multiple circular hole sizes and arrangements. However, changing the diameter of a single circular hole also changes the size of the specimen. In the case of a distribution involving multiple circular holes, they increase the area and number of defects without keeping the size of the defect as the only variable. To better compare the influence of the number and arrangement of circular hole defects with different sizes on the mechanical features of rock mass, the model with a 15 mm diameter circular hole defect was selected. In order to ensure that the total area of the circular hole defects was the same, four numerical models with two horizontally arranged circular holes, two vertically arranged circular holes, three horizontally arranged circular holes, and three vertically arranged circular holes, respectively, were built and the distance between each circular hole and the model boundary was kept constant. The effect of the hole defects’ number and arrangement on the rock samples’ mechanical properties was analyzed under the same defect area. The established model is shown in Fig 11.

### Comparative analysis of stress-strain

**Fig 12:**
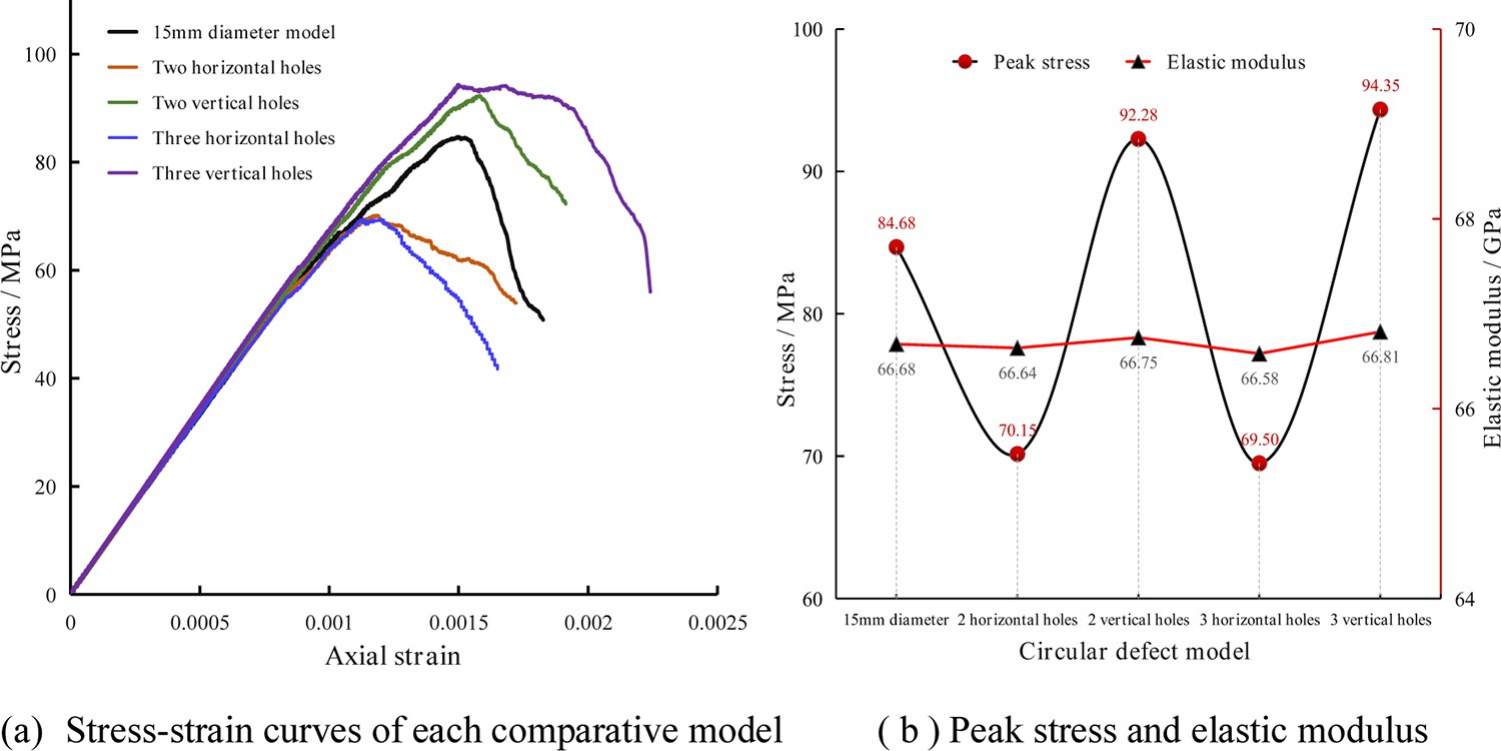
Stress-strain curves, peak stress, and elastic modulus changes of the models with two and three circular holes. (a) Stress-strain curves of each comparative model, (b) Peak stress and elastic modulus.

The stress-strain curves of the models with two and three-circular hole defects were compared to those of the model with a circular hole diameter of 15 mm. The resulting stress-strain curves, peak stress, and elastic modulus changes are shown in Fig 12.

Fig 12 indicates that the peak stresses of the models with the two and three horizontally arranged circular holes are about 70.15 MPa and 69.50 MPa, respectively. The peak stresses of the models with the two and three vertically arranged circular holes are about 92.28 MPa and 94.35 MPa, respectively. Compared with the uniaxial compressive strength of the model with the 15 mm diameter circular hole, the compressive strength of the model with the horizontally arranged circular holes is reduced, and the uniaxial compressive strength of the model with the vertically arranged circular holes is increased. There is a tendency to increase with the number of holes. The elastic modulus of each comparative model shows almost no change compared to the model with the 15 mm diameter circular hole. It can be seen that under the condition of the same defect area, the number and arrangement of hole defects significantly influence the rock mass’s compressive strength but have little effect on the elastic modulus.

Comparing the stress-strain curve and peak stress in Fig 12 with those of the various models with different defect sizes in Fig 6 shows that the peak stress of the comparison model falls between the model with the 10 mm diameter round hole defect (68.01 MPa) and the model with the 20 mm diameter round hole defect (100.14 MPa). This indicates that although the different numbers and arrangements of the hole defects have a more significant impact on the strength of the rock, the size (area) of the defects also has a more pronounced effect on the strength of the rock.

### Comparative analysis of the meso-mechanical characteristics

**Fig 13:**
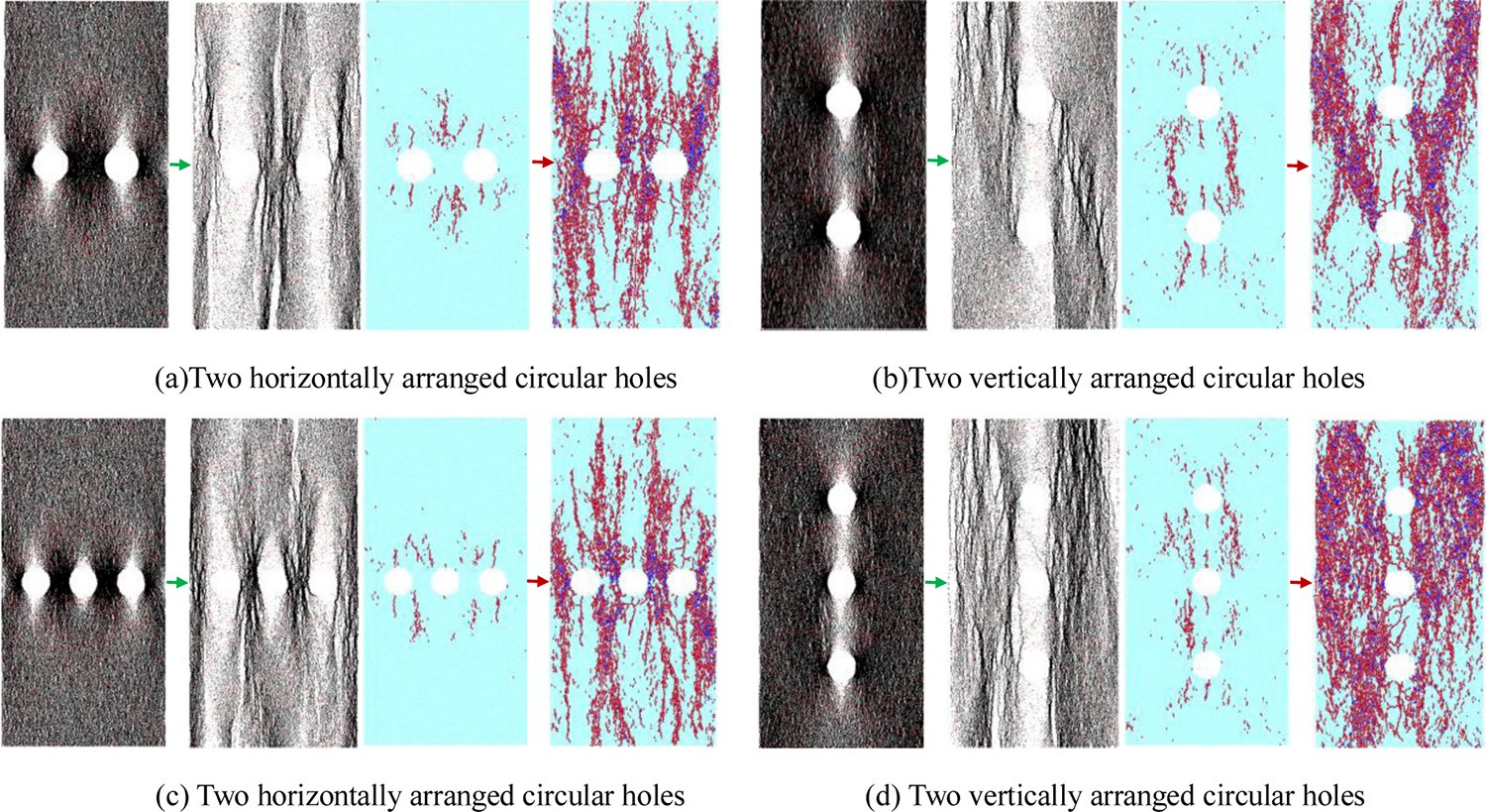
Contact force chain and crack evolution characteristics of the models with two and three circular holes. (a) Two horizontally arranged circular holes, (b) Two vertically arranged circular holes, (c) Two horizontally arranged circular holes, (d) Two vertically arranged circular holes.

The contact force chain and crack evolution characteristics of the pre-peak and post-peak stages of the model with two and three horizontally aligned circular holes were selected for comparison and exploration. The contact force chain and crack evolution characteristics of these models are shown in Fig 13. The contact force chain pictures are respectively at 50% peak stress and 60% peak stress in post-peak stage, and the crack evolution pictures are respectively at 70% peak stress and 60% peak stress in post-peak stage.

Comparison of the contact force chains and crack evolution of the above models with the model containing 15 mm diameter circular holes in Figs 7 and 9 show that the compressive force chains in the model with the horizontally aligned circular holes are still concentrated on the left and right sides of each circular hole. Due to the close horizontal distance between the circular holes, the compressive force chains overlap with each other, making the concentration of the compressive force chain even more concentrated. In the failure stage of the model, the compressive force chain is mainly manifested as a vertical band-like distribution along the circular holes. The cracks in the post-peak stage are also concentrated along the horizontal circular holes where they expand and penetrate. The primary coalescence cracks form a vertical multi-stripe distribution in the model and are also the rock sample’s main failure path and failure form. The superposition and concentration of the contact force chain and the distribution of the through cracks directly cause the models with the horizontally arranged holes to reduce in strength and be more easily damaged. In the models with the vertically distributed circular hole defects, although the vicinity of the circular holes is also the concentrated area of the force chain, the diameter of the circular holes decreases and the distance between the circular hole defects relatively increases. Under uniaxial compression, the lateral dimensions on both sides of the rock sample are equivalent to the bearing stress increase so that the compressive force chain and new cracks spread from the vicinity of the hole to the left and right sides of the model. As a result, the contact force chains and cracks after model failure are mainly concentrated in the dense rock mass on both sides of the model, enhancing the model’s damage-bearing capacity with the vertically arranged circular holes. Compared to the models with the single circular hole and the horizontally arranged circular holes, the failure morphology is significantly altered.

Although the above models keep the defect area constant, when the number and distribution of the holes change, it greatly influences the contact force chain, the distribution of crack evolution, and the stress and strain in the model. It can be seen that the size (area) and the number and distribution of the hole defects can all affect the mechanical features of a rock mass. Ensuring the uniqueness of the defect size as a variable is the scientific basis for studying the effect of defect size on the mechanical features of rock masses. In addition, further studies on defective rock masses under the influence of various factors such as a different number of defects, arrangement of defects, and the shape of defects also need to be carried out in more depth to provide more theoretical support for research on the deformation, failure, and reinforcement of defective rock masses.

## Conclusions

Taking defect size as the only variable, numerical models of limestone containing circular hole defects of different sizes were used to analyze their effects on the rock’s microscopic damage and mechanical properties. The effect of the number and arrangement of hole defects on the models’ mechanical properties is also discussed while keeping the defect area constant. Conclusions are as follows:

1. The UCS and elastic modulus of the limestone rock mass are gradually reduced as the size of the circular hole defect increases. There is a negative correlation between the defect size and the mechanical strength of the rock sample.
2. The vicinity of the circular hole defect is the concentrated area of the contact force chain, and the defect’s size greatly influences the evolution of the force chain inside the rock sample. Before the peak strength, the concentration and distribution of the force chain near the hole are positively correlated with the size of the hole defect; the larger the defect size, the more obvious and larger the force chain concentration near the hole. In the post-peak failure stage, the larger the circular hole defect size, the sparser the distribution of force chains above and below the circular hole in the middle of the model, and the larger the range of the low-stress region above and below the central circular hole in the vertical stress field.
3. The size of the circular hole defect affects the entire process of crack initiation, propagation, and coalescence process. As the size of the circular hole defect increases, the crack initiation stress in each model gradually decreases, making the rock sample more likely to be damaged and deformed. The larger the size of the circular hole, the more the number of cracks that are developed at the upper and lower vertices of the circular hole in each model, and the higher the degree of crack coalescence after failure. The overall number of cracks produced after model failure is roughly negatively correlated with the size of the hole defect. The larger the defect size, the progressively smaller the total number of cracks and the number of tensile and shear cracks, and the more likely the model will fail.
4. When the size (area) of the circular hole defects is the same, the number and arrangement of the defects significantly influence the strength, failure mode, and meso-mechanical characteristics of the rock mass but have little effect on the elastic modulus of the rock mass. The compressive strength of the models with horizontally arranged circular holes is significantly reduced. However, the superposition of the compressive force chain and the expansion of cracks are mainly concentrated between the horizontal holes, which causes the rock sample to be easily damaged. In the models with circular holes oriented vertically, the compressive force chain and new cracks spread from the vicinity of the holes to both sides of the models, increasing the compressive strength. Ensuring that the size of the circular hole defect is the only variable is a prerequisite for studying the effect of circular hole defect size on the mechanical properties of the rock mass.
